# The rotational atherectomy with a guide extension catheter for calcified and tortuous lesions in left anterior descending artery: a case report

**DOI:** 10.1186/s12872-021-02167-3

**Published:** 2021-07-30

**Authors:** Taichi Kato, Masashi Fujino, Kensuke Takagi, Teruo Noguchi

**Affiliations:** Department of Cardiovascular Medicine, National Cerebral and Cardiovascular Center, 6-1, Kishibe-shimmachi, Suita, Osaka 564-8565 Japan

**Keywords:** Guide extension catheter, Calcified nodule, Tortuous, Rotational atherectomy, Case report

## Abstract

**Background:**

The interventional treatment of calcified lesions with severe tortuosity in the left anterior descending artery (LAD) was challenging and the report of rotational atherectomy with mother-and-child technique has been scarce.

**Case presentation:**

An 84-year-old woman was hospitalized for non-ST-segment acute coronary syndrome. Coronary angiography revealed a calcified nodule in the LAD. During rotational atherectomy of the calcified and tortuous lesion in the proximal LAD, eccentric cutting due to wire bias nearly caused perforation. The burr seemed to protrude from the contrast media during angiography. Intravascular ultrasound imaging revealed that extremely eccentric ablation almost reached the adventitia. We successfully ablated the distal calcified nodule by preventing proximal overcutting of the tortuous lesion with support from a guide extension catheter, i.e., the mother-and-child technique, followed by the deployment of the drug-eluting stent. The patient was discharged without chest symptoms and no symptom recurred during 12-month follow-up.

**Conclusion:**

This case demonstrated that safe ablation of a calcified nodule located distal to a tortuous and calcified lesion in the proximal LAD with the mother-and-child technique.

**Supplementary Information:**

The online version contains supplementary material available at 10.1186/s12872-021-02167-3.

## Background

It is often difficult to pass a device through a severely calcified lesion. Therefore, a debulking device is used. When there are proximal vessel tortuosity and a calcified lesion, using a debulking device is sometimes challenging because there is a risk that wire bias might cause coronary artery to be unexpectedly cut away during debulking. There have been reports on the usefulness of rotational atherectomy and orbital atherectomy combined with guide extension catheter (GEC) use in patients with right coronary artery tortuosity [1–3]. However, there have been no reports on the usefulness of intravascular ultrasound (IVUS) imaging for avoiding coronary perforation during rotational atherectomy in a tortuous, calcified left anterior descending coronary artery (LAD). Herein, we report a case successfully treated using rotational atherectomy supported by a GEC, the “child” catheter, for a calcified nodule complicated with a calcified and tortuous lesion in the proximal LAD. IVUS imaging helped us in our decision-making for this strategy. We also describe the device compliance status chart required for this strategy.

## Case report

**Fig. 1:**
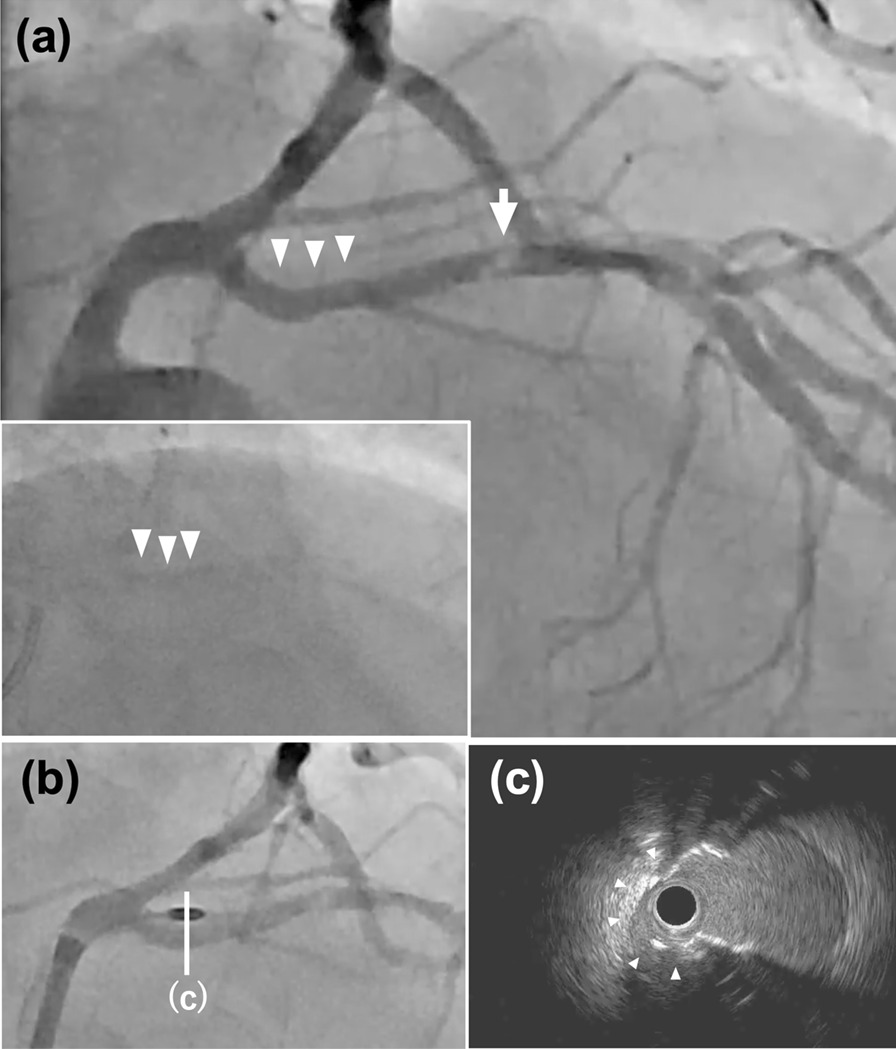
Coronary angiography and intravascular ultrasound (IVUS) imaging. **a** Coronary angiography before rotational atherectomy showing a tortuous calcified lesion (arrowheads) and a calcified nodule (arrow). **b** Rotational atherectomy. Drilling with a 1.5-mm eccentric burr (arrow). **c** IVUS view (**b**, white line) showing eccentric medial cutting at 9 o’clock (arrowheads)

An 84-year-old woman with chest pain was referred to our hospital’s emergency department. Her past medical history included hypertension, hypercholesterolemia, and diabetes mellitus. She developed new and worsening chest pain over 2 weeks. On presentation, her vital signs were stable, and her chest pain had resolved. Electrocardiography showed no significant ST changes. Troponin T was elevated at 0.016 ng/L (reference, < 0.014 ng/L). Transthoracic echocardiography showed evidence of preserved left ventricular ejection fraction without local asynergy. Due to symptoms and cardiac biomarker elevation, the patient was suspected of having non–ST-segment elevation myocardial infarction. She underwent emergency coronary angiography, which showed a severely calcified nodule (Fig. 1a, arrow) distal to a tortuous and calcified lesion in the proximal LAD (Fig. 1a, arrowheads, Additional file 1: Video S1). We decided to perform percutaneous coronary intervention for the calcified LAD lesion.

The treatment was performed via the left radial artery with a 7-Fr guiding catheter (SPB3.5SH; Asahi Intecc). A guidewire (SION blue; ASAHI Intecc) was passed through the stenosis with the aid of a microcatheter (Zizai; Terumo). After guidewire crossing, we failed to pass the lesions with an IVUS catheter (Altaview; Terumo). A rotational atherectomy system was prepared because device delivery was difficult. We used a Zizai microcatheter to change to another guidewire (Rotawire floppy; Boston Scientific). Rotational atherectomy with a 1.5-mm burr was performed several times at 180,000 rpm for the proximal LAD lesion (Fig. 1b, Additional file 2: Video S2). The burr was constricted because it was not directly coaxial to the calcified nodule as a result of the proximal tortuous and calcified lesion. Angiography revealed that the calcified proximal portion was ablated eccentrically due to wire bias. The burr seemed to protrude from the contrast media (Fig. 1b, Additional file 3: Video S3). IVUS imaging revealed extremely eccentric ablation of the proximal calcified lesion; more than half of the media at the 9 o’clock position was cut away (Fig. 1c, arrowheads, Additional file 4: Video S4). The eccentric ablation almost reached the adventitia.

**Fig. 2:**
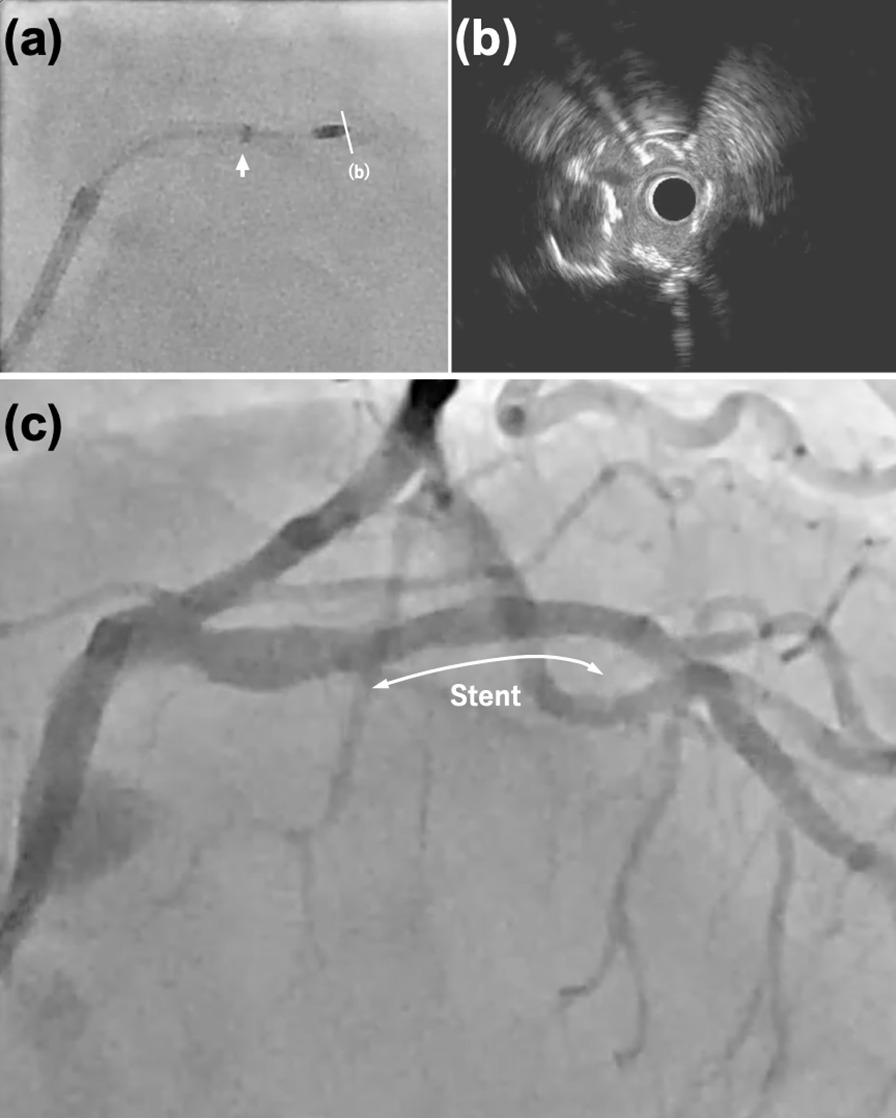
Coronary angiography and intravascular ultrasound (IVUS) imaging. **a** Rotational atherectomy supported by the Guidezilla II (arrow). **b** IVUS view (**a**, white line) showing a cracked calcified nodule at 7 o’clock. **c** Final result after stent (bidirectional arrows) deployment

IVUS imaging helped us decide to perform the “mother-and-child” technique for these lesions. We advanced a GEC (7-Fr Guidezilla II; Boston Scientific) with an inner lumen diameter of 1.60 mm beyond the tortuous part using the balloon surfing technique. This mother-and-child technique successfully enabled coaxial ablation of the calcified nodule (Fig. 2a, Additional file 5: Video S5) without injury at the site of the proximal lesion. During ablation, flow to the left circumflex artery was maintained, and the patient’s vital signs were stable. After ablation, IVUS showed that the calcified nodule at the 7 o’clock position was scored. It was expected to be well dilated (Fig. 2b, Additional file 6: Video S6). After exchanging the guidewire for the SION blue wire, the ablated LAD lesion with a calcified nodule was dilated with a 2.5 mm × 15 mm non-compliant scoring balloon (Scoreflex NC; OrbusNeich) to expand the lesion with lower pressure than the conventional non-compliant balloon and prepare the lumen before stenting [4]. A 2.5 mm × 16 mm drug-eluting stent (Synergy XD; Boston Scientific) was deployed. Final angiography showed excellent results (Fig. 2c, Additional file 7: Video S7). The patient had restored her symptom after the procedure and no symptom recurred during 12-month follow-up.

## Discussion and conclusion

During treatment of complex lesions, IVUS is helpful for assessing lesion characteristics and improving clinical outcomes [5, 6]. A detailed evaluation of the lesion could contribute to the prevention of complications. Since the burr did not reach the calcified nodule coaxially due to wire bias in this case, we performed angiography, which revealed that the burr seemed to protrude from the contrast media. IVUS showed this hazardous condition that the adventitia had been almost cut down. Further cutting was predicted to be dangerous. For complex lesions that are difficult to treat, meticulous evaluation using IVUS can prevent complications.

We could bail out using a GEC to prevent further excavation and perforation of the proximal lesion. To reduce the risk of unexpected vascular damage caused by wire bias, GEC use may be valuable from the start of the ablation for highly tortuous lesions, as in this case. There have been previous reports of GEC use for retrieving a trapped burr [7], but this use is also potentially useful. Another concern is the possibility of ischemia while passing the GEC through the left main coronary artery. If the left main coronary artery vessel diameter is sufficient, the procedure can be performed without adverse hemodynamic effects. In Japan, the available burr size is from 1.25 to 2.25 mm. Since the 7-Fr Guidezilla II (Boston Scientific) with an inner lumen diameter of 1.60 mm was used, the diameter of the burr could be used up to 1.5 mm. Guidezilla II has the largest inner lumen among commercially available GECs (Table 1). Therefore, the Guidezilla II catheter is recommended for rotablation with the mother-and-child technique.

**Table 1:** Size of the guide extension catheter and the rotaburr that can be used with the “mother-and-child” technique

Guide extension catheter	Size (Fr)	Inner lumen diameter (mm)	Outer lumen diameter (mm)	Maximum possible burr size (mm)	Maximum burr size within recommended range* (mm)
Guidezilla II (Boston Scientific)	6.0	1.45	1.70	1.25	1.25
	7.0	1.60	1.85	1.50	1.50
	8.0	1.83	2.11	1.75	1.50
Guideliner V3 (Lifeline)	5.5	1.30	1.64	1.25	N/A
	6.0	1.42	1.70	1.25	1.25
	7.0	1.57	1.90	1.50	1.25
Guideplus (Nipro)	6.0	1.33	1.76	1.25	N/A
Hikyaku (Kaneka)	6.0	1.48	1.75	1.25	1.25
Telescope (Medtronic)	6.0	1.42	1.71	1.25	1.25
	7.0	1.57	1.90	1.57	1.25

*The recommended burr size is 0.1 mm smaller than the inner lumen diameter of the guide extension catheter

In Japan, we can use the Rotablator (Boston Scientific) or the Diamondback 360° Coronary Orbital Atherectomy System (Cardiovascular System) as debulking devices. The Diamondback 360°, which can change bias via pushing and pulling, has been thought to be an effective device for highly tortuous lesions. It was reported that the Diamondback 360° with a GEC was useful for treating angulated or tortuous arteries [8]. However, we used the Rotablator with GEC support in this case; first, we were concerned about the passability of the Diamondback 360° for the lesion with a calcified nodule that Altaview could not pass and secondly, the Diamondback 360° had a risk of lethal coronary perforation or dissection due to unassessed atherosclerotic plaque morphology in the target lesion.

Our case involved safe ablation of a calcified nodule located distal to a tortuous and calcified lesion in the proximal LAD with GEC support, i.e., the mother-and-child technique. This technique might help patients with tortuous and calcified lesions regardless of lesion location when the bias of a rotational burr increases the risk of coronary perforation.

## Supplementary Information

**Additional File 1. Video S1**. Coronary angiography before rotational atherectomy.

**Additional File 2. Video S2**. Rotational atherectomy for the proximal tortuous calcified lesion in the left anterior descending artery.

**Additional File 3. Video S3**. Coronary angiography during rotational atherectomy of the proximal lesion.

**Additional File 4. Video S4**. Intravascular ultrasound view. Eccentric medial cutting at 9 o’clock.

**Additional File 5. Video S5**. Rotational atherectomy supported by the Guidezilla II catheter.

**Additional File 6. Video S6**. Intravascular ultrasound view. Cracked calcified nodule at 7 o’clock.

**Additional File 7. Video S7**. Final result after stent deployment.

## Data Availability

All relevant data supporting the conclusions of this article are included within the article.

## Abbreviations

LAD: Left anterior descending artery
GEC: Guide extension catheter
IVUS: Intravascular ultrasound
